# Management of lercanidipine overdose with hyperinsulinaemic euglycaemia therapy: case report

**DOI:** 10.1186/1757-7241-19-8

**Published:** 2011-01-20

**Authors:** George Hadjipavlou, Aqib Hafeez, Ben Messer, Tom Hughes

**Affiliations:** 1 John Radcliffe Hospital, Headley Way, Headington, Oxford, OX3 9DU, UK

## Abstract

This case report describes the first reported overdose of the dihydropyridine calcium channel blocker (CCB) lercanidipine. A 49 yr old male presented to the Emergency Department 3 hrs after the ingestion of 560 mg of lercanidipine. In the department he had a witnessed seizure within 15 minutes of arrival attributed to the overdose. Following immediate recovery of consciousness after the seizure, he had refractory hypotension and bradycardia which failed to respond to fluid resuscitation, glucagon therapy, and intravenous calcium. He went on to require vasopressor support with noradrenaline and was treated with high dose insulin therapy which was successful in achieving cardiovascular stability. Vasopressor therapy was no longer required within one half life of lercanidipine, and the total stay on intensive care was one day before transfer to a ward.

Calcium channel blocker overdose is an uncommon but life-threatening overdose. Treatment for severe toxicity is similar to b-blocker overdose. Hypotension is treated with intravenous fluid therapy, intravenous calcium and possibly glucagon with vasopressor or inotropic support as required. Atropine is used to attempt reversal of bradycardia. High doses of intravenous insulin with intravenous dextrose as required (hyperinsulinaemic euglycaemia or HIET), has also been successfully reported. Experimental animal data suggests that HIET is of benefit and potentially superior to fluid therapy, calcium, glucagon and potentially vasopressor therapy. HIET effectively and sustainably reverses hypotension, bradycardia and improves myocardial contractility and metabolism. Current advice in calcium channel blocker overdose is to begin therapy early in toxicity, starting with a 1.0 IU/kg insulin bolus followed by an infusion of 0.5 IU/kg/hr of insulin and dextrose as required titrated to clinical response.

## Background

This is to the authors' knowledge the first report of an overdose on the dihydropyridine calcium channel blocker lercanidipine. A pubmed search conducted on July 2010 using the words "lercanidipine," "overdose," and "poisoning" revealed no results. There are no known reported cases on Toxbase[1].

Calcium channel blocker (CCB) overdose is an uncommon overdose, that can cause severe systemic toxicity. Calcium channel blockers are either dihydropyridine or non-dihydropyridine blockers. Dihydropyridine blocker overdose results in arterial vasodilation and a reflex tachycardia, but in high enough doses, the peripheral selectivity is lost and it can also affect the myocardium causing arrhythmias, bradycardia and negative inotropy [2].

Lercanidipine differs to the other dihydropyridine calcium channel blockers in that it has a half life of 10.5 hrs [3], a long time to peak effect compared to other calcium channel blockers when exposed directly to arterial tissue [4] (nifedipine 8.8 mins, verapamil 5.8 mins, lercanidipine 57-63 mins, and amlodipine 106.7 mins) and its effect on the vasomotor reactivity of smooth muscle is slowest to recover following exposure and washout [4] (Percent of response when compared to a standard baseline stimulus applied 95 mins following drug wash out; nifedipine 102.2%, verapamil 84.3%, amlodipine 78.9% and lercanidipine 23.5-47.5%). Compared to the other calcium channel blockers, it is considerably more potent in its action. In rat mesenteric artery samples the concentration to produce a 50% maximal response for S lercanidipine is 22x, 73x and 340x less than amlodipine, nifedipine and verapamil respectively [3]; with R lercanidipine being between nifedipine and verapamil. This means that compared to other calcium channel blockers an overdose on this drug would take longer to reach maximal effect and longer still for its toxic effects to wear off.

When assessing a calcium channel blocker overdose, the time of ingestion, the amount ingested, the release preparation and the type of blocker are important. Basic clinical variables obtained during a standard Airway, Breathing, Circulation and Disability assessment protocol may reveal tachypnoea, brady/tachycardia, hypotension, signs of cardiac failure or changes in conscious state. An electrocardiogram is needed to identify PR prolongation and any bradyarrhythmias. A glucose measurement may reveal hyperglycaemia [2]. Laboratory tests investigating renal function, electrolytes, liver function, and full blood count should be requested in addition to a screen for concomitant overdose.

Treatment requires the use of intravenous fluids, glucagon and or calcium infusions, vasopressor therapy. Treatment with high dose insulinaemic euglycaemia therapy (HIET) has also been reported. Reports of the success of calcium and glucagon in reversing toxic side effects have been documented in case reports and case series of overdoses on verapamil, diltiazem and nifedipine [5-7]. Success with HIET has been documented more recently [8]. In the case of lercandipine overdose described below, a number of therapies were tried to achieve cardiovascular stability. Success was only achieved with HIET. We discuss therefore the importance of this therapy and its possible benefits over other treatments with this described case adding to the body of evidence supporting its use.

## Case Presentation

A 49 year old male with a previous history of hypertension was brought into the Emergency Department by ambulance. He was found asleep with a suicide note and an empty box of tablets five hours after having been last seen. On arrival, his observations were: Glasgow Coma Score 15, respiratory rate 18, heart rate 78 bpm and blood pressure 98/64 mmHg. Fifteen minutes after arrival he suffered a 30 second tonic-clonic seizure with spontaneous recovery of consciousness. Subsequent assessment revealed a bradycardia of 50-60 bpm, and a blood pressure of 77/40 mmHg with maximal head down tilt.

An initial blood gas revealed a normal pH, sodium, potassium, calcium, lactate and a glucose level of 6.9 mmol.l^-1^. A brief history revealed that this gentleman had ground up and then ingested 28 tablets of 20 mg lercanidipine slow release used to treat his hypertension, potentially up to 3 hours prior to presentation. The overdose time was approximately deduced from evidence of activities at the scene. Non-significant doses of zopiclone (max 15 mg), diazepam (max 20 mg) and eprosartan (max 1.2 g) were also ingested. It was unclear exactly how much of the co-ingestants were taken due to limited information of the tablet size at the time. The maximum possible dose was determined from knowing the patient had taken up to 2 tablets of each co-ingestant and by using the British National Formulary for the maximum tablet dose availability. No gastrointestinal decontamination was performed as is our standard practice for overdoses presenting more than one hour after ingestion.

Despite 2 litres of 0.9% sodium chloride given over 5-10 minutes, his systolic blood pressure remained below 80 mmHg with severe diastolic hypotension of 23 mmHg with maximum head down bed tilt. He was transferred immediately to the resuscitation room where he then received 10 mls of 10% calcium chloride through a large bore peripheral cannula over 15 minutes, and 10 mg of glucagon given over 10 minutes according to advice from Toxbase. Serial ECGs did not demonstrate any cardiac conduction defects. The PR interval was normal and the heart rate 50 to 60 bpm.

**Figure 1:**
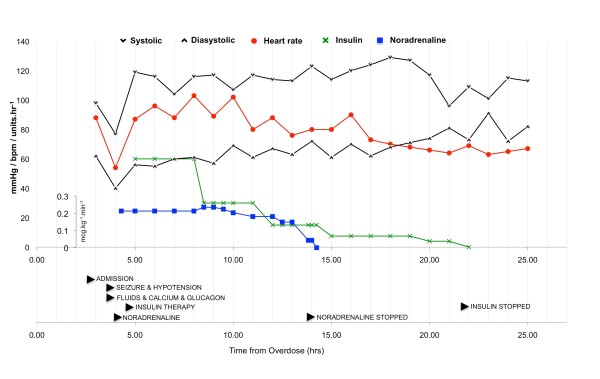
**Time course of heart rate and blood pressure with response to therapies**. Infusions are plotted as curves with rates on the y-axis. Single bolus therapies are named only; doses can be found in the text in the case description.

He remained hypotensive following glucagon and calcium. At this point post seizure, the National Poisons Information Service was consulted and advised further boluses of calcium with regular monitoring of serum calcium levels. High dose insulin therapy at 0.5 IU/kg/hr was also recommended (hyperinsulinaemic euglycaemia) if toxicity was severe. He was started immediately on a noradrenaline infusion targeting a mean arterial pressure of more than 70 mmHg and following this high dose insulin HIET (60 IU/hour; 0.5 IU/kg/hour where weight = 120 kg) in the resuscitation room without a bolus dose. A glucose infusion was started at 100 mls/hour of 20% dextrose. He was subsequently transferred to intensive care. No further boluses of calcium nor glucagon were given due to the initial rapid response to HIET. The initial noradrenaline dose was 0.235 microg/kg/min; noradrenaline was required for a total of 9-10 hours during which time it was gradually weaned. Hyperinsulinaemic euglycaemia therapy was continued for 8 hours after. Figure 1 illustrates the treatments tried over the course of the admission plotted with haemodynamic parameters. Blood glucose levels ranged from 5.6-14.7 mmol/l, and a total of 60 mmol/l of potassium was given. Insulin therapy was gradually weaned and his blood pressure remained stable. He was transferred to the medical ward the following day and discharged home two days after admission following psychiatric assessment.

## Discussion

Treatment for this specific case required a number of the therapies described for significant CCB overdose. However cardiovascular stability was only achieved following institution of hyperinsulinaemic euglycaemia therapy (HIET). A review of the literature reveals that HIET is potentially superior to other treatment options for calcium channel blocker overdose. An in depth review has been made by Lheureux et al [9] and the authors direct the reader to this review for more information. A brief review of the human and animal literature is provided here.

This case study demonstrates the rapid haemodynamic stabilization achieved with HIET. This has been seen in both animal models of calcium channel blocker overdose and in a limited number of human case studies. Kline et al, [10] demonstrated using a canine model of intravenous verapamil overdose, that HIET therapy was superior to intravenous fluids, epinephrine therapy, and glucagon therapy. Twenty-four anaesthetised dogs received verapamil at 0.1 mg/kg/min until a 50% reduction in blood pressure or AV dissociation for greater than 30 minutes was achieved. Four groups of six dogs received either fluid therapy, glucagon, epinephrine or HIET. Outcome was mortality at 240 mins. All those receiving fluid therapy died within 85 minutes. 50% of the glucagon group, and 66% of the epinephrine group had died at 240 mins. There were no deaths in the HIET group. Kline et al, [11] later demonstrated in a canine model that verapamil toxicity renders the heart dependent on carbohydrate metabolism, and HIET was superior to glucagon, adrenaline and calcium chloride in producing the largest increases in the myocardial use of lactate following verapamil toxicity. This was associated with a better outcome compared to the other therapies.

There are few case studies documenting the efficacy and safety of HIET in human CCB overdose. Yuan et al, [8] describes four cases of verapamil overdose, all suffering with hypotension, bradycardia and acidosis that was reversed with HIET when a combination of calcium, glucagon and epinephrine therapy had failed to maintain cardiovascular stability. HIET restored and maintained a significant rise in both systolic and diastolic blood pressure. In one case, an echocardiogram demonstrated an improvement in ejection fraction from 10 to 50% 3 hrs following institution of HIET. This particular case study presented with hypotension, bradycardia and a seizure. Like the case studies, both calcium and glucagon were administered, but these therapies were not continued. Instead HIET was instituted immediately and with observable therapeutic effect. Haemodynamic stability was achieved within 1 hour of the onset of treatment (Figure 1). In other case studies [8], response to HIET has been variable (20 mins to 2 hrs). Though noradrenaline was also started, it was rapidly reduced (4 hours after initiation of HIET) whilst haemodynamic stability was maintained on HIET.

The authors believe that stability was achieved by HIET and not noradrenaline. Withdrawal of noradrenaline was started four hours after HIET initiation; which is not enough time for the serum level of lercanidipine to drop significantly. Assuming a half life of 10.5 hrs [3], there would been more than 50% of the drug remaining compared to when HIET was started; and 40% of the total overdose remaining when noradrenaline was stopped. Unfortunately serum drug levels are not available, and the human toxic range of lercanidipine is unknown. A pubmed search using the items "lercanidipine, LD50, toxicity, lethal" revealed no valuable results. The lethal dose 50% for oral lercanidipine occurs in male rats, mice and dogs at 939 mg.kg^-1^, 622 mg.kg^-1 ^and >300 mg.kg^-1 ^[12]. The single toxic dose therefore in this case works out as 4.67 mg.kg^-1 ^(weight = 120 kg and dose 560 mg). This is considerably different to the animal literature.

Based on a review of current available cases, and experimental data, Lheureux et al, [9] recommends treatment with HIET in CCB overdose with administration of a 1.0 IU/kg IV insulin bolus followed by an infusion of 0.5 IU/kg/hr insulin. This should be co-administered with a high concentration of glucose (some adult requirements range from 20-30 g/h) with half hourly checks of glucose levels. Therapy should be guided by clinical response and haemodynamic stability with the aim to achieve withdrawal of vasoactive agents whilst maintaining cardiovascular stability. Treatment should be started early, as there is a small body of evidence to suggest that benefits of HIET are lost if initiation is late[9]. Cumpston K et al describe 3 cases where HIET failed in diltiazem overdose [13]. In one case HIET was initiated 5.5 hrs post ingestion of 32 g of diltiazem (though this is an extreme overdose). The patient did not survive. The remaining two cases describe failure of HIET to achieve haemodynamic stability when initiated at 13 hrs and 9.5 hrs post ingestion of 7.2 g and 3.4 g of diltiazem; with one case dying and the other remaining in a persistent vegetative state. In the successful case series described by Yuan TH et al [8], the time to initiation of HIET was 3-7 hrs in those cases where it could be ascertained from the information provided. It is understandably hard to make a fair comparison from the lack of numbers and the range of drugs and doses overdosed on. For the above described case, HIET was initiated within 7 hrs of suspected overdose time.

## Conclusions

This is the first reported case of overdose on the dihydropyridine calcium channel blocker lercanidipine. Management in this case report agrees with animal literature and the limited studies available that HIET is potentially superior to other treatments for CCB overdose and its early administration is most likely to be beneficial.

## Abbreviations

HIET: hyperinsulinaemic euglycaemia therapy

## Competing interests

The authors declare that they have no competing interests.

## Authors' contributions

All authors have read and approved the final manuscript. GH was the junior physician involved in the care of the patient in the Emergency Department and the author of the article. AH was the supervising physician involved in the care of the patient in the Emergency Department, and proof read and reviewed article for content. BM was the physician involved in the care of the patient in the Intensive Care Unit. In addition to proof reading and reviewing the article for content, he also reviewed some of the primary material. TH was the supervising consultant of the patient during their time in the Emergency Department. TH proof read and reviewed article for content. All authors contributed to revisions and reviewer responses.

## Consent

Written informed consent was obtained from the patient for publication of this case report and any accompanying images. A copy of the written consent is available for review by the Editor-in-Chief of this journal.
